# Twenty‐Five Years of the Environmental Stress Response and the Enduring Power of Yeast in Stress Biology

**DOI:** 10.1002/yea.70028

**Published:** 2026-05-21

**Authors:** Audrey P. Gasch

**Affiliations:** ^1^ Department of Medical Genetics, Center for Genomic Science Innovation University of Wisconsin‐Madison Madison Wisconsin USA

**Keywords:** environmental stress response, Msn2/Msn4, stress, transcriptome

## Abstract

All organisms must be able to sense and respond to adverse environments, especially those that threaten cellular integrity. The age of genomics clarified the breadth and specificity of cellular stress responses, including in free‐living microbes directly exposed to a changing environment. The environmental stress response (ESR) in *Saccharomyces cerevisiae* was among the first responses defined at the transcriptome‐wide level as a common program triggered by diverse types of stress. Since its original publication over 25 years ago, many studies have explored the role, regulation, and evolution of the ESR and underlying principles of stress defense. This perspective reviews the history of the ESR, recent insights and perspectives into its purpose and regulation, and remaining questions in stress biology primed for the power of yeast experimentation.

## Summary

- Transcriptomic analysis in budding yeast revealed fundamental insights into stress biology.
- Many of the principles of stress defense are conserved across species.
- Exciting questions about stress biology and cellular defense are primed for investigation.

## 1 Introduction

Each cell is an intricate system of interconnected processes operating within a range of tolerated parameters. Failure of individual enzymes, pathways, and molecular interactions can catalyze a chain reaction of cellular defects, thereby causing widespread cellular dysfunction across many important features in the cellular system. Thus, cells have evolved defense strategies to buffer and protect myriad features of the system, known as homeostasis, or adjust the system to new setpoints required for specific environments, known as allostasis (Ramsay and Woods 2014). Changes to the external environment can directly perturb internal homeostasis for free living microbes, hence they have evolved intricate mechanisms to protect the most important features of the system (e.g., genome integrity, cellular structures, redox balance, etc.). Many of the strategies used by microbes are conserved in multicellular organisms as well, highlighting the universal goals of cellular protection.

*Saccharomyces cerevisiae* has been an important model to understand cellular mechanisms of stress defense. Before the age of genomics in the late 1990s, researchers had discovered several transcription factors that induced genes following heat shock, DNA damage, and oxidative stress (Ingolia et al. 1982; M. J. Miller et al. 1979; Moye‐Rowley et al. 1989; Slater and Craig 1987; Yoshimoto et al. 2002). These responses were highly specific to particular insults, reinforcing the notion that cells respond to specific stressors with specialized defense strategies. But around this time, the paralogous transcription factors Msn2 and Msn4 (Msn2/4) were identified as “general stress” factors, shown to induce a set of genes in response to multiple types of stress (Boy‐Marcotte et al. 1998; Estruch and Carlson 1993; Korziuk 1977; Martínez‐Pastor et al. 1996; Moskvina et al. 1998; Wieser et al. 1991). Through painstaking molecular work, researchers identified ~40 stress‐induced proteins that required Msn2/4 activity. Because cells lacking *MSN2/4* are sensitive to extreme doses of heat, osmotic, or oxidative stress (Martínez‐Pastor et al. 1996), it was proposed that Msn2/4 are part of a “general” defense response that protects cells from adversity. However, the relative contribution of condition‐specific versus “general” defense strategies remained unclear in the early years.

### 1.1 History of the ESR and the Early Days of Transcriptomics

Publication of the *S. cerevisiae* genome sequence in 1996 (Goffeau et al. 1996) brought in a new era of whole‐genome investigation, including the development of then‐revolutionary DNA microarrays to characterize transcriptome‐wide responses. Initial microarray experiments reported thousands of transcript measurements over dozens of conditions, revealing genes whose expression was induced or repressed upon environmental shifts. But with only paltry knowledge of gene functions and little ability to exploit the new world of large data sets, insights were limited. It took the arrival of data analysis and visualization tools (Eisen et al. 1998) for the first glimpses into systems‐level cellular responses to be realized, unveiling fundamental insights that now seem given: that sets of genes change in expression together, that those genes often encode proteins of similar functions, and that similarly expressed genes can point to regulatory mechanisms that control their changes (Brown and Botstein 1999).

These tools ushered in a period of exploration of the unknown. But most of the original explorers were off on their own continents, studying isolated responses to individual conditions. It was not until data analysis software packages allowed data sets to be easily compared on a global scale that the true power of Big Data became apparent. Comparing and contrasting responses informed on both the biology of each response and shared consequences of the conditions. Increasing the breadth and diversity of data sets being compared increased the resolution with which expression modules could be discerned. “More Data is Good” became the motto of the Pat Brown and David Botstein labs collaborating in the early days of transcriptomics (Brown and Botstein 1999).

Once data sets from seemingly disparate conditions were compared, a surprising result emerged: despite different cellular challenges posed by each stimulus, much of the transcriptomic responses were, to a first approximation, very similar. Another insight emerged as the first clustering software came onto the scene: clustering gene expression patterns without regard to the directionality of change grouped commonly induced genes together with commonly repressed genes. These gene groups displayed near mirror‐image expression changes across conditions, with the same dynamics, magnitude, and responsiveness, suggesting a shared regulatory program. From this, the ESR was born.

### 1.2 Identity of the ESR Genes

**Figure 1:**
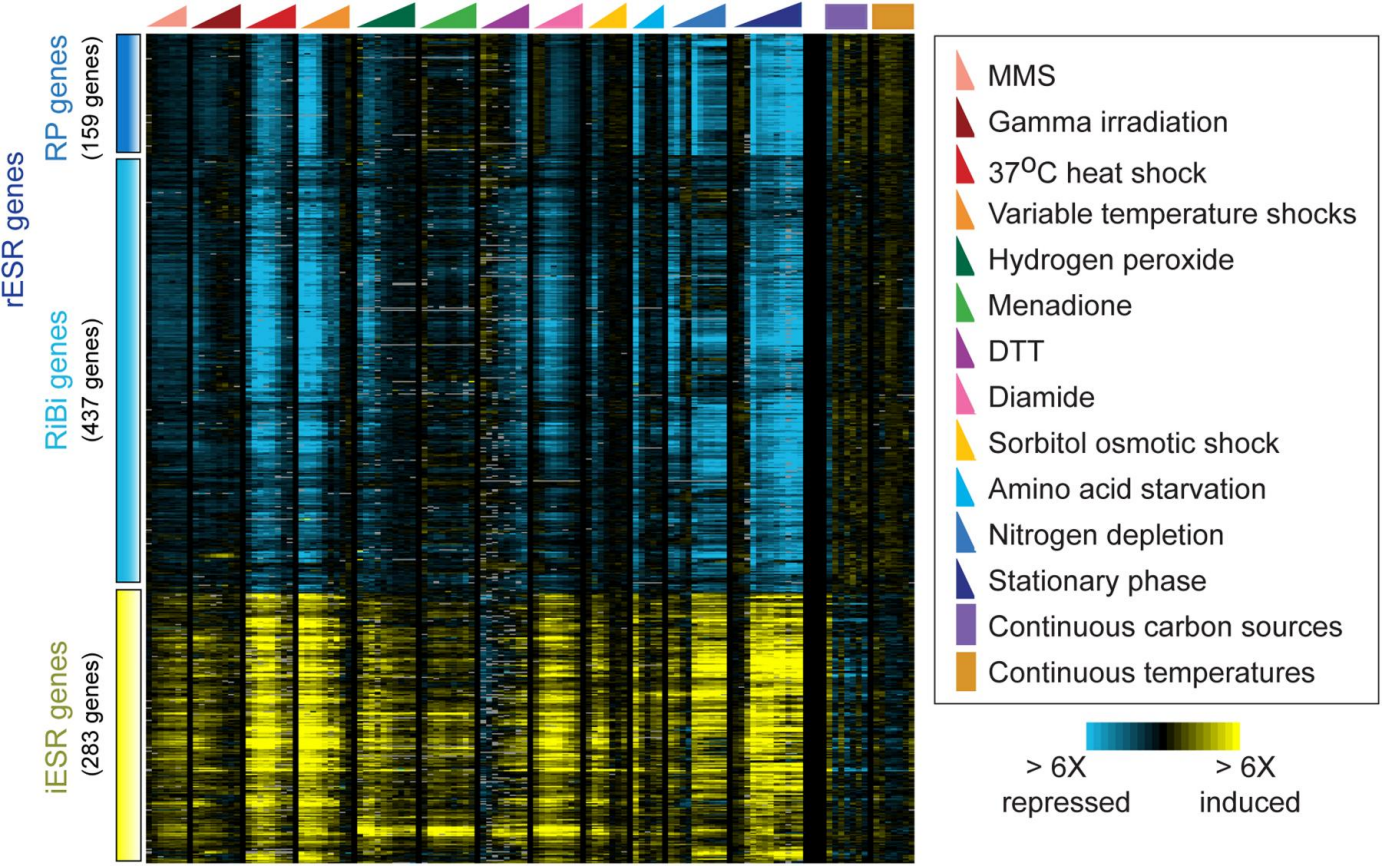
ESR gene expression changes during environmental shifts. Log_2_(fold‐change) in expression is shown for ESR genes (rows) as measured in individual samples (columns). Yellow indicates increases and blue indicates decreases in mRNA abundance compared to unstressed cells, according to the key. Each triangle represents a timecourse experiment (or dosage response for variable temperature shock) whereas rectangles indicate continuous growth on different carbon sources or at different temperatures. Data from (Gasch et al. 2000, 2001).

The original analysis compared timecourse responses to diverse environmental shifts, including heat shocks, oxidative stress, reducing agents, high osmolarity, carbon and nitrogen starvation, and other sudden shifts (Gasch et al. 2000). A similar study was published investigating an overlapping set of environments (Causton et al. 2001). The ESR was defined by hierarchical clustering of this rich dataset, which identified several clusters of genes with similar patterns of expression across dozens of conditions (Figure 1). The ESR gene set should not be considered a hard and fast definition of genes that are (or are not) part of the response. Rather, their expression changes serve as a signature of sudden environmental decline, since the ESR is not activated when cells are returned to “optimal” growth conditions. The program includes 283 genes whose mRNA abundance increases during stress (induced “iESR” genes), many of which are regulated in part by Msn2/4, along with 596 genes whose mRNA abundance is reduced (repressed “rESR” genes). The ESR gene set was updated slightly in 2017 based on refinements to the yeast genome annotation, for a total of 879 genes in the program (https://gasch.genetics.wisc.edu/).

Functions of the encoded proteins immediately suggested a stress response. iESR genes encode proteins involved in a variety of stress defense processes, including redox balance, protein folding and degradation, carbohydrate and energy metabolism, trehalose and glycogen biosynthesis, and more (Gasch 2003). The group includes genes encoding both positive and negative regulators of the ESR, suggesting feedback mechanisms of control (Portela and Rossi 2020; Segal et al. 2003; Smith 1998). In contrast, the rESR group is subdivided into two clusters with slightly different expression dynamics: 159 mostly‐ribosomal protein (RP) genes in the RP cluster and the remaining 437 genes involved in ribosome biogenesis, transcription and translation, and other growth‐related processes in the PAC/RiBi group, so named for the enrichment of upstream PAC promoter element (GATGAG) (Dequard‐Chablat et al. 1991) and genes involved in ribosome biogenesis (Gasch et al. 2000; Jorgensen et al. 2004). rESR genes are enriched for essential genes, many of which are highly expressed in the absence of stress and required for rapid growth (Gasch 2007).

iESR and rESR genes show parallel but opposite changes in expression across diverse types of stress, and both the magnitude and dynamics of their changes co‐vary across conditions. Some stresses like sudden heat shock provoke large and rapid expression changes, whereas other shifts as well as gradual environmental changes produce muted responses. Furthermore, larger doses of the same condition produce larger changes in mRNA, as measured in bulk cultures (Gasch et al. 2000). Because of the dose‐responsive nature of ESR expression changes, the program is often used as a rheostat to indicate the level of stress experienced by cells. It is important to note, however, that there are exceptions where ESR activation does not correlate with growth rate or other markers of cellular stress. For example, some yeast mutants are extremely slow growing but do not activate the ESR (Kemmeren et al. 2014). A number of stresses like DNA damage lead to complete arrest of division, yet only mild ESR activation (Gasch et al. 2001). The reason for these exceptions is not always clear, but offers caution in interpreting cell experiences based solely on transcript abundances.

### 1.3 Why Does the Cell Mount the ESR?

On first inspection, the known functions of the characterized ESR genes suggested a hypothetical purpose for mounting the program: iESR gene products may defend against the imposing stress while rESR repression responds to, or perhaps causes, retarded growth as cells acclimate. However, neither hypothesis is fully born out.

#### 1.3.1 Role of iESR Induction

The yeast gene‐deletion project was the first whole‐genome mutation library before the ease of CRISPR mutagenesis, and it generated a wealth of information about yeast gene functions (Giaever et al. 2002; Winzeler et al. 1999). Surprisingly at the time, most genes induced by various stress treatments are not required to survive those stressors (Birrell et al. 2002; Giaever et al. 2002). Furthermore, actively growing cells lacking Msn2 and Msn4 (Msn2/4) show a major defect in iESR induction when exposed to single‐stress treatments yet do not show a survival defect (see more below) (Berry et al. 2011; Berry and Gasch 2008; Kocik et al. 2026). Consistently, protein synthesis is not required for short‐term survival of acute fungicidal stresses (Berry et al. 2011; Berry and Gasch 2008; Guan et al. 2012; Hall 1983; Ho et al. 2018). Together, these results strongly suggested that transcript induction is dispensable for surviving single‐stress treatments.

However, transcriptional induction, including iESR activation, is essential to survive tandem stress treatments. Acquired stress resistance is a phenomenon in which cells exposed to a mild dose of a one stress become resistant to what would otherwise be a lethal dose of the same or a different secondary stress treatment (Święciło 2016). Although there are certainly condition‐specific relationships involved in cross‐stress protection, a common theme is that Msn2/4 play a role (Berry and Gasch 2008; Kandror et al. 2004; Liang et al. 2023; Scholes et al. 2024). Actively growing *msn2∆msn4∆* cells can readily survive single‐stress treatments, but they often have major defects acquiring tolerance to subsequent stresses, depending on the precise conditions (Berry and Gasch 2008). In contrast, cells with artificial activation of *MSN2/4* are resistant to a variety of insults (Estruch and Carlson 1993; Sasano, Haitani, et al. 2012; Sasano, Watanabe, et al. 2012; Watanabe et al. 2009), confirming that iESR induction *before* stress exposure bolsters survival. Thus, gene induction, including induction of iESR genes, can serve a preparatory role for impending stress, one that can accommodate the delay required to make new mRNAs and proteins. In fact, it is our perspective that this phenomenon explains why *msn2∆msn4∆* cells were originally scored as sensitive to very high doses of stress. Some studies investigated stress tolerance in saturated cultures where Msn2/4 are already induced by the diauxic shift, whereas others scored the small subset of wild‐type cells with very high stress tolerance due to stochastic activation of Msn2/4 (Boy‐Marcotte et al. 1998; Martínez‐Pastor et al. 1996). According to this hypothesis, in both cases *msn2∆msn4∆* cells would show a defect when exposed secondarily to other stressors. It remains possible that Msn2/4 are required to survive single‐stress treatments under specific circumstances, separable from an acquired‐stress function.

#### 1.3.2 Purpose of rESR Repression

Transcriptomic analysis was also early applied to understand growth‐rate responses. Initial studies observed that ESR activation correlates with reduced cell division and growth during nutrient restriction (Brauer et al. 2008; Castrillo et al. 2007; O'Duibhir et al. 2014; Regenberg et al. 2006; Slavov et al. 2012). This led to the suggestion that perhaps the ESR was not an active response to stress, but rather an indirect byproduct of reduced growth (which requires lower ribosome production to fuel the growing daughter cell). While RP production often does parallel growth rate, the relationship is not universal (e.g., translation inhibitors lead to slower growth rate but increased abundance of RP and RiBi transcripts [Cheng and Brar 2019; Ho et al. 2018; S. Levy and Barkai 2009; Santos et al. 2019; Shamsuzzaman et al. 2023]). More importantly, cells already arrested in their division and biomass production still mount the ESR when exposed to acute stress. It is our perspective that the rESR is not merely a passive byproduct of growth‐rate changes, but rather an active response to cellular stress.

We proposed that the primary purpose of dynamic rESR repression is to help the cell produce new proteins from induced transcripts. Most ribosomes in rapidly growing cells are actively involved in translation (Warner et al. 2001); if so, this would leave little capacity to make new proteins during a rapid response. Since many rESR transcripts are highly translated during active growth, they are a prime consumer of translating ribosomes. Nonetheless, during acute stresses studied to date, increases in mRNA correlate with increases in the corresponding proteins (Csárdi et al. 2015; Lahtvee et al. 2017; M. V. Lee et al. 2011; Liu et al. 2016; Vogel and Marcotte 2012). A major question had been the source of ribosomes needed to translate hundreds of newly expressed transcripts.

We proposed that ribosomes are released as rESR mRNAs drop in abundance. Much of this model comes from detailed analysis of transcriptomic, proteomic, and translational changes triggered by sodium chloride treatment, which induces both osmotic and ionic stress. Like other stresses, acute salt exposure activates transient iESR induction and rESR repression before transcripts acclimate to new steady‐state levels (Kocik et al. 2026; M. V. Lee et al. 2011). Unlike the transient changes of induced mRNA, encoded proteins increase progressively in abundance before stabilizing (M. V. Lee et al. 2011). Modeling and simulations support a model in which the transient over‐shoot of iESR transcripts accelerates protein production, we proposed through mass‐action competition for ribosomes that become available when rESR transcripts drop. Consistent with this model, cells lacking RiBi repressors Dot6 and Tod6 (Dot6/Tod6) fail to repress their targets during salt treatment, and the resulting mRNAs remain bound to ribosomes (Ho et al. 2018). (In fact, recent work shows that mRNAs made after stress, including rESR transcripts in the *dot6∆tod6∆* mutant, are marked for translation whereas pre‐existing mRNAs are translationally silenced (Glauninger et al. 2025; Zedan et al. 2025; Zid and O'Shea 2014)). Aberrantly abundant rESR transcripts continue to bind ribosomes, which we argued explains the delayed ribosome binding of iESR transcripts, delayed production of their encoded proteins, and delayed acquisition of subsequent severe‐peroxide tolerance (Ho et al. 2018; Kocik et al. 2026; M. V. Lee et al. 2011). While it remains to be seen if this model holds for all stress treatments, it provides an explanation for the tight coupling of iESR induction with rESR repression across many conditions, due to intricate coregulation of the two modules (Gasch 2003; Ho and Gasch 2015; Kocik et al. 2026).

The coupling of iESR and rESR expression may also be important to balance the cost of mounting the response. Cells lacking *MSN2/4* grow equally to wild‐type in the absence of stress, but they grow *faster* than wild type during non‐lethal exposures to single‐stress treatments including heat, ethanol, acidic, or basic pH (Kocik et al. 2026). We put forth a model in which the cost of the Msn2/4 response is likely due to the burden of making hundreds of new transcripts and proteins, a cost that can be significant (Bragg and Wagner 2009; Kafri et al. 2016; Lahtvee et al. 2017). We argue that this cost is accommodated by rESR repression: cells lacking *DOT6/TOD6* grow normally before, but abnormally slowly after, multiple stresses—unless *MSN2/4* are also deleted, since a quadruple mutant regains wild‐type growth rates after stress (Kocik et al. 2026). Modeling suggests that the cost of mounting the ESR is a tradeoff for the benefits of acquired stress resistance, which may provide selective pressure to maintain the ESR over evolutionary time to aid in acclimating to dynamic environmental changes (Kocik et al. 2026; Liang et al. 2023; Pradhan et al. 2021). Additional research in this area will continue to expand and refine these models.

### 1.4 Regulation of the ESR: Specialized Networks to Activate a Common Response

Although ESR expression changes are common to many stresses, the program is not regulated through a singular mechanism. Instead, ESR activation is customized for each condition through a combination of condition‐specific regulators integrated with multi‐stress‐responsive controls. Regulation of the ESR involves many pathways, transcription factors, and posttranscriptional mechanisms, several of which have been covered in detail elsewhere (Estruch 2000; Gasch 2003; Kocik and Gasch 2022; Lempiäinen and Shore 2009; C. Miller et al. 2011; Shalem et al. 2008; Zencir et al. 2020). Below is a high‐level summary of ESR regulatory principles.

**Figure 2:**
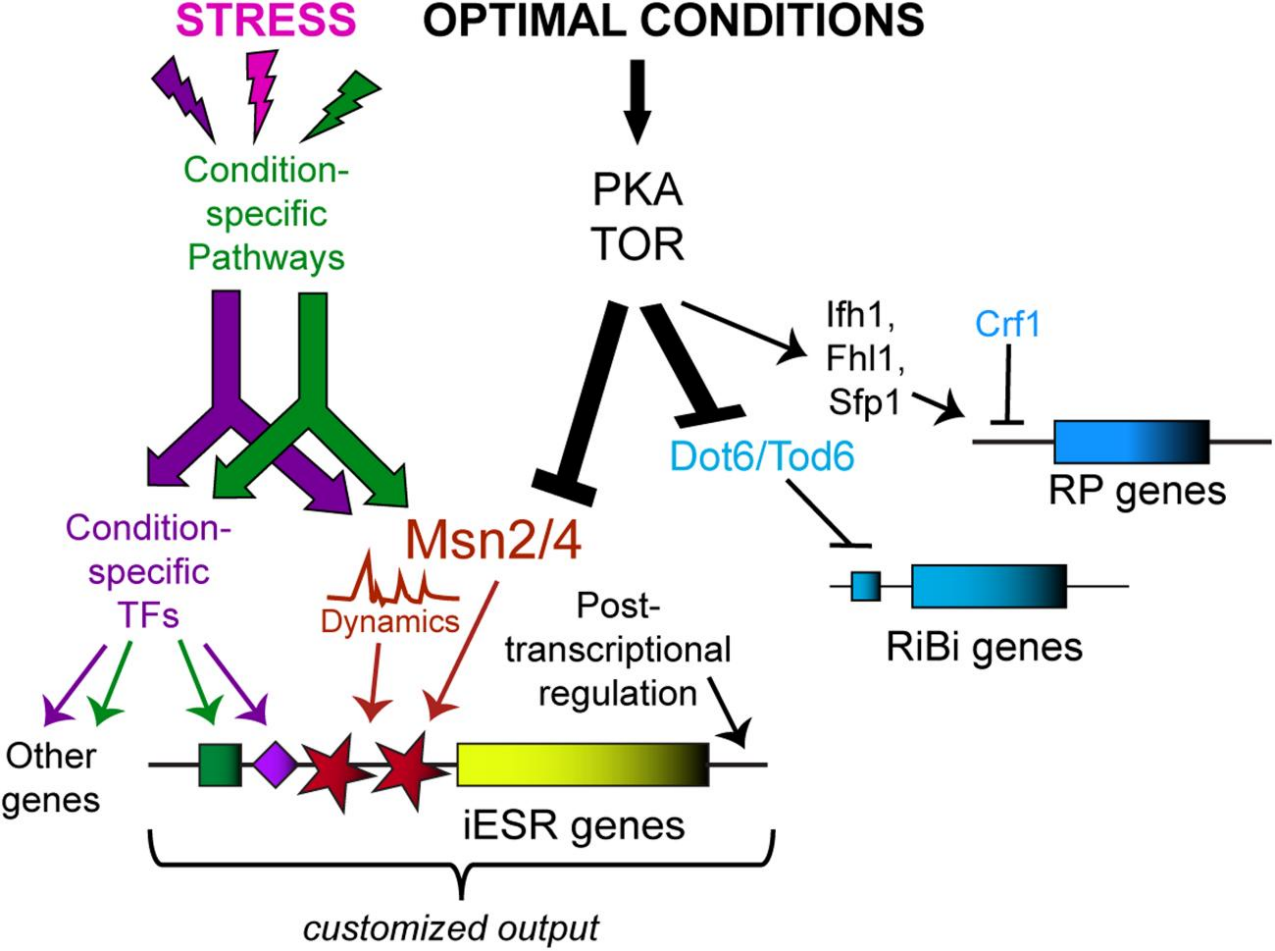
Regulatory themes in ESR control. Regulatory architecture of ESR genes is represented for induced iESR genes and repressed RiBi and RP gene sets represented by boxes. Upstream shapes represent transcription‐factor binding sites. See text for details.

Several pathways and transcriptional regulators play a role under multiple conditions (Figure 2). In optimal environments that support rapid growth, the ESR is suppressed by growth‐promoting RAS/Protein Kinase A (PKA) and TORC1 pathways (Broach 2012; Kocik and Gasch 2022; Rohde et al. 2008). Both PKA and TORC1 can directly phosphorylate Msn2/4 and Dot6/Tod6, rendering them inactive in the cytosol (Beck and Hall 1999; Garreau et al. 2000; Lippman and Broach 2009), while simultaneously activating positive regulators for high rESR gene expression (Jorgensen et al. 2004; Kumar et al. 2022; Martin et al. 2004; Schawalder et al. 2004; Shore et al. 2021). Both PKA and TORC1 are rapidly suppressed during multiple stress treatments, which along with phosphatase action removes positive regulatory signals and relieves inhibitory Msn2/4 and Dot6/Tod6 phosphorylation, which in turn allows the factors to move into the nucleus to modulate transcription (Besozzi et al. 2012; Düvel and Broach 2004; Garmendia‐Torres et al. 2007; Granados et al. 2018; Jacquet et al. 2003; Lippman and Broach 2009; Reiter et al. 2013; Santhanam et al. 2004). This response network functions under a variety of stress conditions and can thus be considered part of the “common” regulation of ESR activation (although Msn2/4 and Dot6/Tod6 paralogs can also play condition‐specific roles [AkhavanAghdam et al. 2016; Berry and Gasch 2008; Chapal et al. 2019; Granados et al. 2018; Lippman and Broach 2009]).

On top of this regulatory backdrop, condition‐specific pathways also contribute to ESR activation, best understood for iESR genes. Each pathway responds to specific sensors that orchestrate specialized responses while contributing to ESR regulation. For example, the HOG1 pathway affects ESR activation during osmotic stress (Capaldi et al. 2008; O'Rourke and Herskowitz 2004), whereas the Protein Kinase C pathway responds to cell structural integrity (Gutin et al. 2019; Hirata et al. 2003). Others include ATM/ATR kinase activated by DNA damage (Gasch et al. 2001), CK2 kinase complex responding to multiple stresses including peroxide or lactic acid stress (Cho and Hahn 2017), Snf1 in response to poor carbon sources (Cho and Hahn 2017; Garreau et al. 2000; Mayordomo et al. 2002), and more (Garreau et al. 2000; P. Lee et al. 2008, 2013; Takatsume et al. 2010). Many of these regulators impact Msn2/4 activation, either through direct phosphorylation or through undetermined mechanisms. In addition to Msn2/4, each pathway activates its own downstream factors to regulate condition‐specific gene sets. The end result is that ESR regulation can be mediated by different upstream kinases under different situations. A benefit of this complicated regulatory system is that it ensures ESR activation without compromising the sensitivity and specificity with which cells can mediate condition‐specific aspects of each response.

Cells also customize iESR activation at the level of transcription. The iESR group is heavily enriched for genes with upstream Msn2/4 promoter elements, where Msn2/4 bind after multiple stress treatments (Elfving et al. 2014; Huebert et al. 2012; Kocik et al. 2026; Teixeira et al. 2023). But subsets of iESR genes are also regulated by stimulus‐specific factors that super‐induce their expression during specific insults. For example, iESR genes involved in oxidative defense are regulated by Msn2/4 in response to many conditions but super‐induced by oxidative‐stress factor Yap1 in response to redox stress (Gasch et al. 2000). A different set of Msn2/4 targets involved in osmolyte production are super‐induced by Hot1 and Sko1 specifically during osmotic shock (Proft et al. 2005; Rep et al. 1999), whereas chaperones in the ESR are super‐induced by Hsf1 during heat shock (Gasch et al. 2000; P. Lee et al. 2008). Each condition‐specific transcription factor controls additional, non‐ESR genes, once again supporting coordinated activation of condition‐specific responses with the common ESR. Undoubtedly, additional regulation details including mechanisms of network‐level integration remain to be fully elucidated.

An open question is if there are additional regulatory inputs to customize rESR repression. RiBi genes show fewer upstream regulatory motifs than the average gene, unlike iESR and RP genes that show more (Kocik et al. 2026). Intriguingly, Msn2/4 bind many rESR promoters after exposure to a number of stresses and are required for full rESR repression, although the mechanistic function awaits functional testing (Chasman et al. 2014; Elfving et al. 2014; Huebert et al. 2012; Kocik et al. 2026; Teixeira et al. 2023).

Condition‐specific behaviors can also arise through emergent properties of the system, and Msn2 has become a model for understanding transcriptional decoding (Granados et al. 2018; Gutin et al. 2019; Hao and O'shea 2011; Stewart‐Ornstein et al. 2013; Sweeney and McClean 2023). Msn2 and Msn4 display different nucleo‐cytoplasmic shuttling dynamics depending on the identity and dose of stress. For example, some conditions lead to perpetual Msn2 nuclear localization, others produce a single nuclear pulse, and still others lead to oscillatory behavior (Bodvard et al. 2011; Hao et al. 2013; Hao and O'shea 2011; Jacquet et al. 2003; Petrenko et al. 2013). Although several studies reported that dynamics are determined by stress identity, results are not consistent across studies and thus regulatory controls are not completely understood. Nonetheless, different dynamic patterns impart distinct transcriptional outputs, depending on promoter architectures and strength of Msn2/4 binding (Hansen and O'Shea 2015, 2016; Hansen and Zechner 2021; Hao et al. 2013; Hao and O'shea 2011; Stewart‐Ornstein et al. 2013; Sweeney and McClean 2023). Msn2 has also become a model to understand how transcription factors scan and recognize DNA. In addition to DNA binding interactions, Msn2 can localize to promoters through protein interactions with its intrinsically disordered domains, independent of DNA binding (Brodsky et al. 2020; Lupo et al. 2023; Mindel et al. 2024; Vidavski et al. 2025). This may explain how Msn2/4 bind to many rESR promoters during stress, even though rESR genes show a dearth of upstream Msn2/4 binding sites (Kocik et al. 2026). Mechanistic details remain to be worked out here.

### 1.5 Remaining Questions and Future Prospects

*S. cerevisiae* was one of the early genomic models for stress biology, owing in part to being the first sequenced eukaryote. Although genomic approaches are now applicable to many organisms, yeast retains its place as an unparalleled system of investigation, especially in stress biology. Many questions remain about genes and pathways cells use to defend against specific stressors, toxins, and disease‐modeling mutations. Since defense strategies are often conserved, understanding stress targets in yeast can have far‐reaching application, from producing toxin‐tolerant chassis microbes for biofuel production (Dunlop 2011; Xu et al. 2022) to improving heat tolerance in sensitive corals (Barshis et al. 2013; Palumbi et al. 2014), to managing drug‐resistant *Candida auris* infections in a changing climate (Cha et al. 2025; Heaney et al. 2020).

Beyond identifying genes and pathways activated by specific conditions, *S. cerevisiae* remains an excellent test bed for understanding higher‐level cellular responses that remain challenging to elucidate in multi‐cellular and nonmodel organisms. Several of these areas are discussed below.

#### 1.5.1 Systems Biology of Stress Defense—How Do Cells Do It?

*S. cerevisiae* is a powerful model for systems biology, which aims to understand quantitatively how thousands of molecules integrate to form a dynamic, living organism. An end‐goal is to be able to predict—comprehensively and with mathematical precision—how a cell will respond at the molecular level to a new stimulus. To accomplish this requires a good understanding of the parts lists, including all genes, encoded functions, and their regulation, as well as knowledge of reaction rates, molecular concentrations, and biophysical properties. *S. cerevisiae* benefits from a deep knowledgebase in this regard, and it is also amenable to both nanoscale and genome‐wide manipulation and characterization. The task now is to integrate this information into dynamic models to address new questions. For example, how do yeast cells set the balance between growth versus defense? What are the intracellular features cells monitor to do this? Is there a single currency of cellular health or many interconnected determinants? How are multiple internal and external signals integrated and interpreted to produce a customized systemic response? What design principles ensure robustness of the system? What are the best strategies to engineer a cell with new properties and responses?

Answering these questions will likely require single‐cell analysis. Like other organisms, *S. cerevisiae* cells show substantial cell‐to‐cell variation in how they respond. Isogenic cells in the same environment can differ widely in growth rate, metabolic state, signaling responsiveness, transcriptional output, and ultimately stress tolerance. For example, some cells in an “unstressed” culture will activate the stress response, either stochastically or in response to some internal stress (Chatterjee and Acar 2018; S. F. Levy et al. 2012; Petrenko et al. 2013; Raser and O'Shea 2004). These cells are often slow growing and stress resistant, suggesting a “bet hedging” strategy for population survival (Bergen et al. 2022; Geiler‐Samerotte et al. 2013; S. F. Levy et al. 2012; Li et al. 2018). Cells experiencing acute stress also vary substantially in their responses. In past work from our lab studying acute salt stress, some cells activate the ESR with large expression changes, while other cells show only mild response (Bergen et al. 2022; Gasch et al. 2017). Roughly 8% of cells activate the Hsf1 chaperone regulon, not known to be activated by salt based on bulk‐culture analysis; a different ~10% activate the Rpn4‐regulated proteasome response (Gasch et al. 2017). These differences indicate that cells in the same environment experience the stressor differently, for reasons that remain to be fully elucidated. Possibilities include differences in cell‐cycle state, metabolic regime, cell age, historical experiences, pre‐stress growth status, and stochastic fluctuations in any number of processes (Bagamery et al. 2020; Berry et al. 2011; Knorre et al. 2018; Pinheiro et al. 2022; Wang et al. 2022). Tracking multiple phenotypes in many single, living cells can reveal phenotypic correlations, implicating causal relationships, and expanding knowledge of the cellular system.

#### 1.5.2 A Little Stress Is Good for You—But How Much Is Too Much?

Acquired stress resistance is observed across species, and thus understanding its governing rules has broad practical application. In some cases, the value is in “breaking” acquired tolerance, for example to prevent acquired resistance of pathogens to host defenses or tumor cells to chemotherapy drugs (Chern and Tai 2020; Frederick et al. 2025; Liang et al. 2023; Pradhan et al. 2021). In other cases, the goal is to bolster stress tolerance, as in surgical or chemotherapy pretreatments that protect healthy cells from damage (Blagosklonny 2023; Chiari and Fellahi 2024; Raffaghello et al. 2008). Several fundamental properties remain poorly defined. What determines how much mild stress is required to provoke resistance? Which mild stresses provide cross‐protection against which other conditions and through what mechanisms? How do these rules vary with the dose, duration, and temporal pattern of mild‐stress pretreatment? And ultimately, how much pretreatment is too much?

Chronic activation of stress responses is clearly deleterious, explaining why many species reserve such responses for tough times. Myriad mammalian diseases and injuries lead to constitutive activation of the Integrative Stress Response (ISR, described below [Costa‐Mattioli and Walter 2020]), perhaps because humans have not adapted for long‐term survival of those stressors. Drugs that suppress constitutive ISR activation have therefore attracted clinical interest to alleviate the effects of a chronic stress response (Bugallo et al. 2020; Chou et al. 2017; Coulson et al. 2024; Jiang et al. 2022; P. J. Zhu, Khatiwada, et al. 2019). But what are the consequences of ablating the stress response without addressing the underlying stress itself? Untangling these tradeoffs, understanding long‐term dynamics, and identifying regime shifts between beneficial and harmful stress responses remain open challenges.

#### 1.5.3 What Are the Conserved Principles of Stress Biology When Underlying Mechanisms Have Evolved?

Many questions can be addressed in a single organism, but driving principles are often only apparent through cross‐species comparison. The reason is that the end goals of cells are frequently conserved even when mechanisms used to achieve them are not. In fact, strong selection to accomplish nonobvious end goals can accelerate evolution, driving mechanisms apart (True and Haag 2001). Thus, what may seem like disparate responses across organisms can nevertheless be driven by the same unifying forces. But what are those forces?

A useful example is to compare yeast and bacterial stress responses. Although few of the regulators are conserved, bacteria like *E. coli* mount a common stress response reminiscent of the ESR. In response to stress, the alarmone metabolite ppGpp is synthesized to suppress transcription of growth‐promoting genes while promoting synthesis of defense proteins (Gourse et al. 2018; M. Zhu, Pan, et al. 2019). Like the ESR, these responses are proposed to be coupled to help balance resource allocation in the growth‐versus‐defense decision (Balakrishnan et al. 2021; Basan et al. 2020; M. Zhu et al. 2024; M. Zhu and Dai 2023). Also akin to the ESR, activation of the response prepares bacteria for future environmental shifts (Balakrishnan et al. 2021; Boutte and Crosson 2013; Gourse et al. 2018; M. Zhu and Dai 2023). ppGpp is synthesized on the ribosome and is controlled by a direct readout of ribosome elongation rate, thereby tracking cellular demand against available resources (C. Wu, Balakrishnan, et al. 2022). Yeast and other organisms lack ppGpp, and thus identifying the mechanisms they use to monitor resource balance remains an active area of investigation.

Stress responses in mammalian cells also share similarities with the ESR. The transcription factor p53 responds to many types of stress (Zhang and Lozano 2017). Like Msn2/4, it resides predominantly in the cytosol under optimal conditions but rapidly translocates to the nucleus after stress, with condition‐ and tissue‐specific dynamical patterns that encode different regulatory outcomes based in part on target‐promoter architecture (François et al. 2026; Purvis et al. 2012; Purvis and Lahav 2013). P53 regulates genes involved in apoptosis, stress defense, and signaling (including regulators of its own response) and can specify cell fate depending on activation kinetics. Interestingly, although p53 does not share sequence similarity with Msn2/4, the two systems share interactions with many of the same upstream pathways in a conserved stress‐activated regulatory network (Ho and Gasch 2015). Mammalian cells also deploy the ISR, again activated by diverse conditions to phosphorylate elongation factor eIF2α (Costa‐Mattioli and Walter 2020; Dever et al. 2023; Harding et al. 2003; Houston et al. 2020). Phosphorylated eIF2α suppresses global translation initiation, including at repressive uORFs to allow selective translation of stress‐activated transcription factors that go on to induce their targets (Young and Wek 2016). Although distinct from the mechanism of the ESR, ISR architecture also enables resource reallocation via reduced global translation coupled with induced production of defense proteins. As seen for the ESR, the mammalian ISR is regulated by different upstream kinases under different stresses (Y. Wu, Zhang, et al. 2022). (It should be noted that yeast also employ a stripped‐down ISR response with a single activating kinase, Gcn2 [Nanjaraj Urs et al. 2025]). These parallels between the yeast ESR and mammalian ISR hint that hard‐wired resource reallocation and conditional deployment of common responses are shared design principles of stress defense. An interesting opportunity is to compare stress responses across broader phylogenetic space to understand how universal demands are met by evolving regulatory systems.

### 1.6 Concluding Remarks

*S. cerevisiae* has been at the forefront of stress biology, providing foundational insights that have advanced study in myriad organisms, conditions, and applications. It will undoubtedly remain in this position, given the exceptional knowledge of its parts, ease and speed of manipulation, and accessibility to the latest innovations in experimental measurement. Many questions in the field of stress biology are ripe for exploration, including mechanisms of memory and inheritance of stress tolerance, the relationship between stress and aging, principles of hysteresis (history‐dependent responses to stress) and hormesis (where a stress can be beneficial or deleterious depending on the dose), and natural variation in stress defense mechanisms within and between species. The questions that can be addressed are limited only by our creativity in asking them.

## Data Availability

Data sharing not applicable to this article as no data sets were generated or analyzed during the current study.
